# Microscopic Analyses of Fruit Cell Plastid Development in Loquat (*Eriobotrya japonica*) during Fruit Ripening

**DOI:** 10.3390/molecules24030448

**Published:** 2019-01-27

**Authors:** Pengjun Lu, Ruqian Wang, Changqing Zhu, Xiumin Fu, Shasha Wang, Don Grierson, Changjie Xu

**Affiliations:** 1 Zhejiang Provincial Key Laboratory of Horticultural Plant Integrative Biology, College of Agriculture and Biotechnology, Zhejiang University, Zijingang Campus, Hangzhou 310058, China; 11316050@zju.edu.cn (P.L.); 3120100252@zju.edu.cn (R.W.); zcq1236@zju.edu.cn (C.Z.); wangshasha1993222@163.com (S.W.); Donald.grierson@nottingham.ac.uk (D.G.); 2 South China Botanical Garden, Chinese Academy of Sciences, Xingke Road 723, Tianhe District, Guangzhou 510650, China; fuxiumin@scib.ac.cn; 3 Division of Plant and Crop Sciences, School of Biosciences, University of Nottingham, Sutton Bonington Campus, Sutton Bonington LE12 5RD, UK

**Keywords:** carotenoids, chromoplast, colour, loquat (*Eriobotrya japonica*), plastid, ultrastructure, DIC (differential interference contrast) microscopy, transmission electron microscopy (TEM)

## Abstract

Plastids are sites for carotenoid biosynthesis and accumulation, but detailed information on fruit plastid development and its relation to carotenoid accumulation remains largely unclear. Here, using Baisha (BS; white-fleshed) and Luoyangqing (LYQ; red-fleshed) loquat (*Eriobotrya japonica*), a detailed microscopic analysis of plastid development during fruit ripening was carried out. In peel cells, chloroplasts turned into smaller chromoplasts in both cultivars, and the quantity of plastids in LYQ increased by one-half during fruit ripening. The average number of chromoplasts per peel cell in fully ripe fruit was similar between the two cultivars, but LYQ peel cell plastids were 20% larger and had a higher colour density, associated with the presence of larger plastoglobules. In flesh cells, chromoplasts could be observed only in LYQ during the middle and late stages of ripening, and the quantity on a per-cell basis was higher than that in peel cells, but the size of chromoplasts was smaller. It was concluded that chromoplasts are derived from the direct conversion of chloroplasts to chromoplasts in the peel, and from de novo differentiation of proplastids into chromoplasts in flesh. The relationship between plastid development and carotenoid accumulation is discussed.

## 1. Introduction

Carotenoids are important plant pigments for coloring plant organs and participate in light harvesting during photosynthesis, as well as serve as precursors for abscisic acid (ABA), strigolactone and aroma compound biosynthesis [1,2]. Carotenoids are also important for human health, as they can reduce the occurrence of certain cancers, as well as cardiovascular and eye diseases [1,3].

In plants, carotenoids are biosynthesized and accumulated in plastids [4,5]. There are several different types of plastids that account for carotenoid accumulation in plant cells, and they all originate from proplastids and can undergo interconversions [4,5,6]. In fruit, carotenoids accumulate in chloroplasts during early developmental stages, and in chromoplasts during later stages.

Some plant mutants accumulate enhanced amounts of carotenoids in fruit, and this has been found to be related to an increased number or size of chromoplasts. A 30% increase in plastid number was found in *high pigment 1* (*hp1*) tomato ripe fruit, and an increase in both plastid numbers and size were observed in *hp2* and *hp3* [7,8,9,10]. In citrus, we previously found that the differences in number, size and shape of chromoplasts were related to the amount of carotenoids accumulated in mature fruit [11,12].

Chromoplasts are derived from fully developed chloroplasts in tomato and pepper fruits during ripening [13,14], from leucoplasts in papaya fruit [15], from amyloplasts in saffron red stigma [16], and from proplastids in *Or* cauliflower curd [17]. In summary, chromoplast biogenesis can occur through several different processes, depending on plant species and tissue types. Fruit of some plants are composed of peel and flesh, and accumulate carotenoids in both tissues. However, whether the developmental processes for chromoplasts in peel and flesh occur by the same or a different mechanisms remains to be resolved. Furthermore, some important details concerning the conversion of chloroplasts to chromoplasts, such as the changes in number and size, are lacking. 

Loquat (*Eriobotrya japonica*), a member of the Rosaceae family, produces carotenoids as the characteristic main pigments in ripe fruit [18,19]. Loquats are generally divided into red- and white-fleshed types according to flesh color, and previously, with fully ripe fruit, we observed huge differences in carotenoid content and composition between these two types, both in peel and flesh [18,19]. We also found differences in the occurrence of chromoplasts in Luoyangqing (LYQ; red-fleshed) and Baisha (BS; white-fleshed) [19]. Differential interference contrast microscopic observation was made previously on loquat fruits [19], but from only one stage—i.e., the fully ripe stage—and the study was qualitative rather than quantitative. Transmission electron microscopic observation was conducted previously, but the stages of fruit spanned from fruitlet to mature fruit [19], and therefore the observation of plastid transition during fruit ripening was not studied in detail. Here, with these two cultivars, and focusing on the transition to ripening, we investigated in detail chromoplast occurrence and development, i.e., changes in plastid number, size and ultrastructure, in peel and flesh tissues during fruit ripening. We found that chromoplasts occurred via two different processes, conversion from chloroplasts in peel cells and de novo differentiation from proplastids in flesh cells. Reduction in plastid size was observed during conversion of chloroplast to chromoplasts, and this suggested that the conversion process involves reconstruction.

## 2. Results and Discussion

### 2.1. Anatomical Changes of Plastids during Loquat Fruit Ripening

**Figure 1:**
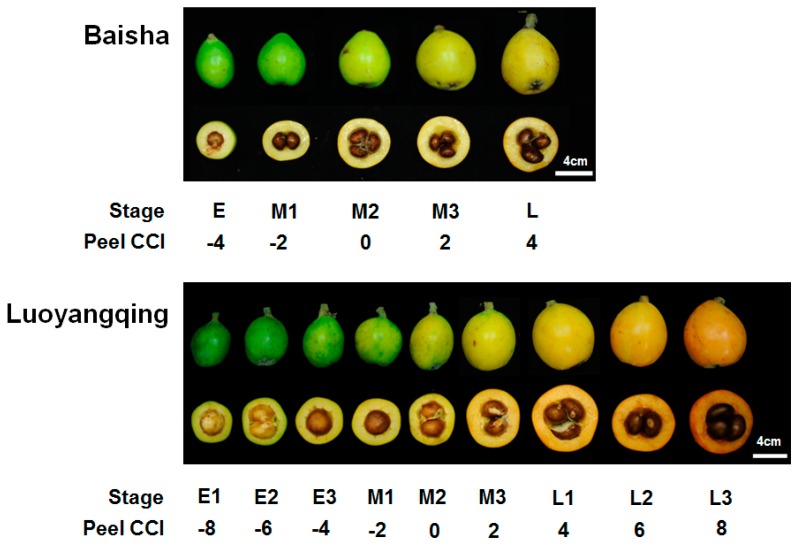
Appearance of Luoyangqing (LYQ) and Baisha (BS) loquat fruits at different maturity stages, as indicated by serial citrus color index (CCI) values for the peel. Bar = 4 cm.

As in many fruits, the peel colour of the loquat turns from deep green to yellow or orange during fruit ripening, and carotenoids accumulate as a result of gradually increased expression of carotenoid biosynthetic genes [19]. The colour transition is accompanied by a gradual increment in citrus color index (CCI), a comprehensive indicator for colour impression [20], and an ideal indicator for fruit maturity level [21]. Here we classified the fruits of BS and LYQ at different maturity stages according to the CCI values of the fruit peel (Figure 1), and aimed to elucidate the general characteristics of plastid changes during fruit ripening, and the differences between two loquats cultivars with hugely different amounts of carotenoids. Fruit enlargement occurred during the ripening of loquat fruits, and the increase in size, indicated by transverse diameter, was around 32% in BS and 51% for LYQ; thus, ripe LYQ fruit are around 20% larger than BS fruit (Figure 1).

**Figure 2:**
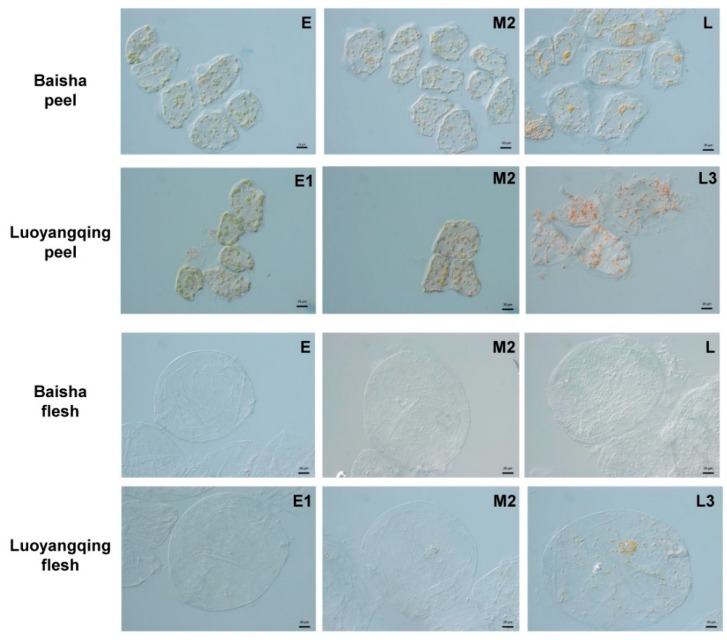
Plastids of Luoyangqing and Baisha loquats in the peel and flesh cells of fruits at early, middle, and late maturity stages under a light microscope. Bar = 20 μm.

Different types of plastids predominated in the peel cells at different maturity stages. Chloroplasts were present at the early and middle stages of ripening, as with turning or breaker stages for other fruits, and those chloroplasts were transformed into light-coloured chromoplasts in peel cells. The chromoplasts in BS were smaller and less coloured than those in LYQ (Figure 2). During late maturity stages, the colour of chromoplasts gradually deepened, reaching a yellow or orange colour in BS and LYQ, respectively (Figure 2). In flesh cells, no plastids could be observed at early maturity stages. Chromoplasts could be observed, however, in LYQ flesh cells at the middle and late stages of ripening. The number of chromoplasts per cell in LYQ mature fruit (L3 stage) was higher in flesh cells than in peel cells, but the flesh cell chromoplasts were a smaller size (Figure 2). In contrast, no chromoplasts or any other type of developed plastid could be observed at any stage in flesh cells of the BS fruit (Figure 2).

**Figure 3:**
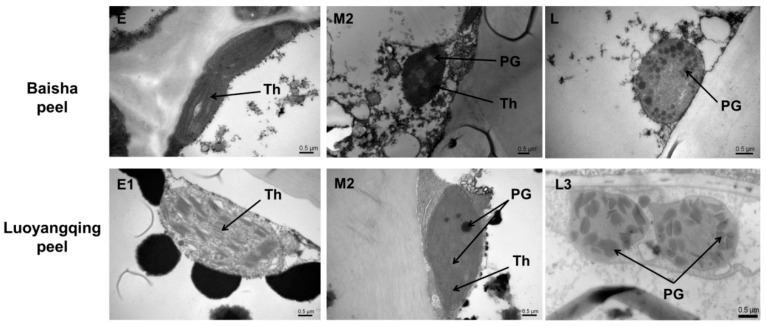
Ultrastructure of plastid of Luoyangqing and Baisha loquats in the peel cells of fruits at early, middle, and late maturity stages. Bar = 0.5 μm. Th: thylakoid; PG: plastoglobule.

The ultrastructure of plastids in BS and LYQ peel cells showed chloroplasts with thylakoid membranes and grana at the early stages of fruit ripening. At the middle stage, the plastids were transformed into chromoplasts, each with some residual thylakoid membrane and a few plastoglobules. At the late stage, the plastids had matured into chromoplasts with more plastoglobules (Figure 3). The shape of chromoplasts in peel cells was to some extent different between the two cultivars, with spindle-like chromoplasts being more commonly observed in LYQ and spherical ones in BS. Larger plastoglobules were found in LYQ compared to BS peel chromoplasts (Figure 2 and Figure 3), and the plastoglobules differed in shape, being stone-shaped in LYQ and a smooth globular shape in BS (Figure 3). The differences in plastoglobules in red fruit peel cells are probably an adaptation for storage of more carotenoids, over half of the amount is β-carotene, and there is over three times the amount in LYQ compared to BS [19]. The ultrastructure of plastids in LYQ flesh cells was similar to that in peel cells (data not shown).

### 2.2. Quantitative Changes in the Abundance and Size of Plastids during Loquat Fruit Ripening

**Figure 4:**
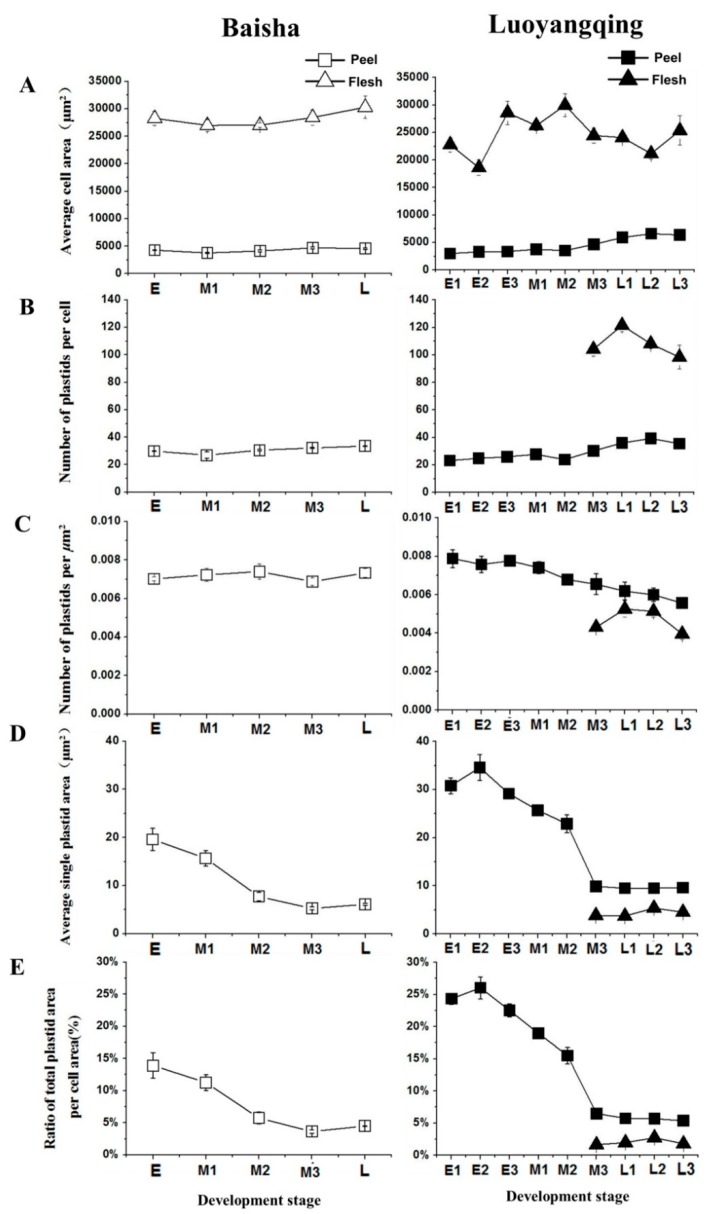
Changes in cell size and plastid number and size during the ripening of Luoyangqing and Baisha loquat fruits. (**A**) Cell size indicated by area; (**B**) plastid population; (**C**) plastid density; (**D**) plastid size indicated by area; (**E**) plastid size (by area) relative to cell size (by area).

Cell size was estimated by measuring average cell area, and flesh cell values were around five times those of peel cells. In general, the size of peel and flesh cells increased slightly during ripening, and this was more obvious for LYQ peel cells (Figure 4A), which was consistent with the greater enlargement of the fruit (Figure 1). Interestingly, although LYQ fruit are larger in size than BS, the size of peel and flesh cells was similar between the two cultivars (Figure 4A), and the number of plastids per cell for LYQ was around five times higher in flesh cells than in peel cells.

During fruit ripening, the number of plastids remained stable in BS peel cells, but the quantity increased steadily, by around half, in LYQ peel cells (Figure 4B). However, for fully ripe fruit, the average number of chromoplasts per peel cell was similar between BS (34) and LYQ (35) (Figure 4B). Unlike the situation for LYQ peel cells, the quantity of chromoplasts in LYQ flesh cells did not change significantly during ripening (Figure 4B). The increased quantity of plastids in LYQ peel cells during ripening is interesting, since it suggests the possibility of either plastid division, especially during the conversion of chloroplasts into chromoplasts, or differentiation of additional undifferentiated plastids, such as proplastids, into mature chromoplasts. On the other hand, the plastids in flesh cells of LYQ arose from the differentiation of undifferentiated plastids, such as proplastids, into mature chromoplasts, since no plastids could be observed at the early stages of maturity (Figure 2 and Figure 4B).

The density of plastids in cells was calculated, indicated by the number of plastids per μm^2^ cell area. In BS peel cells, the density remained stable during ripening, while in LYQ peel and flesh cells, the density declined gradually, by about 30% (Figure 4C). This suggested that the increase in the number of plastids per cell was uncoupled from cell enlargement.

During fruit ripening, not only the type and the number of plastids changed, but the size did as well. Both the average single plastid area and the proportion of total plastid area per cell area declined by around two-thirds for the plastids in peel cells of both cultivars, while no obvious decline was observed for those in LYQ flesh cells (Figure 4D,E). Considering the conversion of chloroplasts to chromoplasts that occurred in peel cells but not in flesh cells, and the decline in size that occurred quickly as the fruit were approaching color break stage (without further change after the color break stage), it can be concluded that the conversion of chloroplasts to chromoplasts is a complicated process, involving both reconstruction and division. A mechanism involving division is supported by the report of a large number of small-sized chromoplasts being derived from a few large chloroplasts during fruit ripening in *suffulta* tomato mutant fruit [22]. Besides the above-described differences in the size of plastids between stages, differences were also observed between cell types and cultivars. Chromoplasts from LYQ peel cells are bigger by about one-fold in terms of area than those from flesh cells, and plastids from LYQ peel cells are bigger than those from BS, with the average area for a single plastid around 10 μm^2^ and 6 μm^2^, respectively (Figure 4D).

### 2.3. Plastid Differentiation and the Relationship between Carotenoid Accumulation and Plastid Development during Loquat Fruit Ripening

Plastids are the main organelles to synthesize and accumulate liposoluble pigments like chlorophylls and carotenoids. Previous studies on tomato *hp* mutants and citrus have suggested the involvement of plastid number and size in regulating carotenoid accumulation in fruits [7,8,9,10], but in general, the studies on characteristics of chromoplasts are still limited to a few plant species. In loquat, the white-fleshed cultivar BS contains only trace amounts of carotenoids, and the development of chromoplasts are impaired in the flesh [19]. The number of carotenoids is over three times less in BS peel than LYQ peel, which explains the lighter colour of the former [19]. However, the detailed information on changes in plastids in the peel during ripening of these two cultivars has not been reported. Here we show that the lighter peel colour of BS, as compared with LYQ, is not due to the lower number, but to the smaller size and special ultrastructure of its chromoplasts (Figure 2, Figure 3 and Figure 4D). Therefore, the increased carotenoid accumulation, as represented by a higher CCI value in LYQ mature fruit, did not result from more chromoplasts, as it does in the *hp* tomato mutants.

Chromoplasts in ripe fruit develop through two main ways: conversion from chloroplasts or development directly from proplastids [5,23]. In this study, a yellow–green mixed color for plastids under light microscopy (Figure 2) and an intermediate type of plastid with plastoglobules under TEM was observed in peel tissues at the breaker stage (M2; Figure 3), suggesting that chromoplasts are derived from chloroplasts in loquat peels. However, it is also probable that some chromoplasts in the peel are derived from de novo differentiation from proplastids, since the number of plastids per cell in LYQ peels increased by around half during fruit ripening (Figure 4B). This possibility needs to be further investigated. In flesh cells, no plastids were observed during early maturity stages (M2 and before), but chromoplasts were found from M3 in LYQ (Figure 2 and Figure 3), indicating that chromoplasts in flesh cells are derived from de novo differentiation from proplastids.

The relationship between chromoplast development and carotenoid accumulation remains a fascinating but frustrating question in plant science. On the one hand, as described previously, the enhanced biogenesis of chromoplasts in *hp* tomato mutants stimulates the accumulation of carotenoids in fruits [7,8,9,10]. On the other hand, the increased biosynthesis of carotenoids through overexpression of *AtPSY* in Arabidopsis root calli resulted in the occurrence of crystalline chromoplasts depositing carotenoid crystals not found in wild types [24]. Recently, in sweet orange, we observed that induced lycopene accumulation via the application of a lycopene cyclase inhibitor to cultured juice vesicle tissue directly affected chromoplast differentiation and structure [12]. According to data obtained in this study, it is probable that the differences in chromoplast characters between two cultivars, such as size and ultrastructure, are an adaptation to differences in the amount of carotenoid accumulated, since the chromoplasts in BS peel are not so deeply coloured as those in LYQ.

## 3. Materials and Methods

### 3.1. Plant Materials

Baisha (BS; white-fleshed) and Luoyangqing (LYQ; red-fleshed) loquat (*Eriobotrya japonica* Lindl.) fruits at different maturity stages were sampled from an orchard in Luqiao, Zhejiang, China. After measurement of peel color, values of CCI were calculated as described below, and the fruits with a colour index around ± 0.2 of an indicated value were selected for further study (Figure 1). BS (white) fruit were classified into five maturity stages: E (short for early ripening), M1 (short for middle ripening 1), M2 (also named breaker stage), M3, and L (short for late ripening), corresponding to CCI values of −4, −2, 0, 2, and 4. LYQ (red) fruit were classified into nine stages: E1, E2, E3, M1, M2, M3, L1, L2, and L3, corresponding to CCI values of −8, −6, −4, −2, 0, 2, 4, 6, and 8. Each stage contained three replicates, with two fruit per replicate.

### 3.2. Color and Fruit Size Measurement

Peel color was measured using a Hunter Lab Mini Scan XE Plus colorimeter (Hunter Associates Laboratory, Inc., Reston, VA, USA). The Commission Internationale de L’Eclairage (CIE) L*a*b* color scale was adopted. L* refers to lightness, which ranges from 0 (black) to 100 (white); positive a* refers to a red–purple color, while negative a* refers to a bluish-green; finally, positive b* refers to yellow, while negative b* refers to blue [25]. CCI was calculated following the formula: CCI = 1000 × a*/(L* × b*), with positive CCI indicating red, negative indicating blue–green, and zero indicating an intermediate mixture of red, yellow, and blue–green [20]. Four random measurements per fruit were made, and the average CCI value was recorded. Fruit size, indicated by transverse diameter, was measured with a Vernier caliper.

### 3.3. Cell Squashing and Differential Interference Contrast Microscopy

The flesh tissue was cut into small pieces with a sterile scalpel blade and fixed overnight in 2.5% glutaraldehyde in 0.1 M phosphate buffer (pH 7.0). Then the fixing solution was removed and 0.1 M Na_2_EDTA was added to the samples, which were then incubated at 60 °C for 2.5 h [22]. The cells were separated by squashing, then observed and photographed with a Zeiss microscope (Carl Zeiss AG, Oberkochen, Germany).

### 3.4. Transmission Electron Microscopy

Juicy sacs were separated and fixed overnight at 4 °C in 2.5% glutaraldehyde in 0.1 M phosphate buffer (pH 7.0). Samples were washed three times (15 min each) with phosphate buffer, then post-fixed with 1% osmium tetroxide (OsO_4_) in 0.1 M phosphate buffer (pH 7.0) for 1–2 h, and washed three times in phosphate buffer. Samples were dehydrated by a linear gradient ethanol series (50%, 70%, 80%, 90%, 95%, and 100%), 15 min for each grade, and infiltrated by absolute acetone for 20 min. Samples were gradually infiltrated with resin (1:1 mixture of absolute acetone and the final Spurr resin mixture for 1 h; 1:3 mixture of absolute acetone and the final resin mixture for 3 h, with Spurr resin mixture overnight). Embedded samples were placed in capsules that contained embedding medium and heated at 70 °C for about 9 h. The samples were stained by uranyl acetate and alkaline lead citrate for 15 min each, and observed in TEM using an Hitachi JEM-1230 (Hitachi, Ltd., Tokyo, Japan).

### 3.5. Measurement of Quantities and Areas of Cells and Plastids

With the cell squashing method, the three-dimensional cells and plastids can essentially be squashed into two dimensions. The areas of squashed cells and plastids can partly represent the proportion of the volume. Pictures of cells from peel and flesh from LYQ and BS were taken at 400× magnification. Measurement of numbers and areas was carried out using ZEN 2012 Lite (Blue edition, Carl Zeiss AG, Oberkochen, Germany). Twenty cells from each fruit were randomly selected and photographed.

## 4. Conclusions

Chromoplasts developed during fruit ripening in the peel cells of both cultivars studied, as well as in LYQ flesh cells, via two different processes: the conversion from chloroplasts in peel cells and de novo differentiation from proplastids in flesh cells. Increased plastid numbers were observed in LYQ peel cells during ripening, and this may indicate the possibility of either plastid division or additional de novo differentiation of proplastids. A reduction in plastid size was observed during the conversion of chloroplasts to chromoplasts, and this suggested that the conversion process is complicated, possibly involving reconstruction. The deeper orange colour (implicating the accumulation of higher amounts of carotenoids) in the peel of LYQ compared to BS was not due to a higher chromoplast number, but was associated with a larger size of both the organelles and the plastoglobules contained within single chromoplasts.

## References

[1] Enfissi E.M.A., Nogueira M., Bramley P.M., Fraser P.D. (2017). The regulation of carotenoid formation in tomato fruit. Plant J..

[2] McQuinn R.P., Giovannonni J.J., Pogson B.J. (2015). More than meets the eye: From carotenoid biosynthesis, to new insights into apocarotenoid signaling. Curr. Opin. Plant Biol..

[3] Fraser P.D., Bramley P.M. (2004). The biosynthesis and nutritional uses of carotenoids. Prog. Lipid Res..

[4] Sun T., Yuan H., Cao H., Yazdani M., Tadmor Y., Li L. (2018). Carotenoid metabolism in plants: The role of plastids. Mol. Plant.

[5] Egea I., Barsan C., Bian W., Purgatto E., Latché A., Chervin C., Bouzayen M., Pech J.C. (2010). Chromoplast differentiation: Current status and perspectives. Plant Cell Physiol..

[6] Lopez-Juez E., Pyke K.A. (2005). Plastids unleashed: Their development and their integration in plant development. Int. J. Dev. Biol..

[7] Cookson P.J., Kiano J.W., Shipton C.A., Fraser P.D., Romer S., Schuch W., Bramley P.M., Pyke K.A. (2003). Increases in cell elongation, plastid compartment size and phytoene synthase activity underlie the phenotype of the *high pigment-1* mutant of tomato. Planta.

[8] Kolotilin I., Koltai H., Tadmor Y., Bar-Or C., Reuveni M., Meir A., Nahon S., Shlomo H., Chen L., Levin I. (2007). Transcriptional profiling of *high pigment-2^dg^* tomato mutant links early fruit plastid biogenesis with its overproduction of phytonutrients. Plant Physiol..

[9] Galpaz N., Wang Q., Menda N., Zamir D., Hirschberg J. (2008). Abscisic acid deficiency in the tomato mutant *high-pigment 3* leading to increased plastid number and higher fruit lycopene content. Plant J..

[10] Enfissi E.M., Barneche F., Ahmed I., Lichtlé C., Gerrish C., McQuinn R.P., Giovannoni J.J., Lopez-Juez E., Bowler C., Bramley P.M., Fraser P.D. (2010). Integrative transcript and metabolite analysis of nutritionally enhanced *DE-ETIOLATED1* downregulated tomato fruit. Plant Cell.

[11] Peng G., Wang C.Y., Song S., Fu X.M., Azam M., Grierson D., Xu C.J. (2013). The role of 1-deoxy-d-xylulose-5-phosphate synthase and phytoene synthase gene family in citrus carotenoid accumulation. Plant Physiol. Biochem..

[12] Lu P.J., Wang C.Y., Yin T.T., Zhong S.L., Grierson D., Chen K.S., Xu C.J. (2017). Cytological and molecular characterization of carotenoid accumulation in normal and high-lycopene mutant oranges. Sci. Rep..

[13] Harris W.M., Spurr A.R. (1969). Chromoplasts of tomato fruits. II. The red tomato. Am. J. Bot..

[14] Egea I., Bian W., Barsan C., Jauneau A., Pech J.C., Latché A., Li Z.G., Chervin C. (2011). Chloroplast to chromoplast transition in tomato fruit: Spectral confocal microscopy analyses of carotenoids and chlorophylls in isolated plastids and time-lapse recording on intact live tissue. Ann. Bot..

[15] Schweiggert R.M., Steingass C.B., Heller A., Esquivel P., Carle R. (2011). Characterization of chromoplasts and carotenoids of red-and yellow-fleshed papaya (*Carica papaya* L.). Planta.

[16] Grilli Caiola M., Canini A. (2004). Ultrastructure of chromoplasts and other plastids in *Crocus sativus* L. (Iridaceae). Plant Biosyst..

[17] Paolillo D.J., Garvin D.F., Parthasarathy M.V. (2004). The chromoplasts of mutants of cauliflower (*Brassica oleracea* L. var. *botrytis*). Protoplasma.

[18] Zhou C.H., Xu C.J., Sun C.D., Li X., Chen K.S. (2007). Carotenoids in white- and red-fleshed loquat fruits. J. Agric. Food Chem..

[19] Fu X.M., Kong W.B., Peng G., Zhou J.Y., Azam M., Xu C.J., Grierson D., Chen K.S. (2012). Plastid structure and carotenogenic gene expression in red- and white-fleshed loquat (*Eriobotrya japonica*) fruits. J. Exp. Bot..

[20] Zhou J.Y., Sun C.D., Zhang L.L., Dai X., Xu C.J., Chen K.S. (2010). Preferential accumulation of orange-colored carotenoids in Ponkan (*Citrus reticulata*) fruit peel following postharvest application of ethylene or ethephon. Sci. Hort..

[21] Olmo M., García J.M. (2000). Nondestructive methods to evaluate maturity level of oranges. J. Food Sci..

[22] Forth D., Pyke K.A. (2006). The *suffulta* mutation in tomato reveals a novel method of plastid replication during fruit ripening. J. Exp. Bot..

[23] Barsan C., Zouine M., Maza E., Bian W., Egea I., Rossignol M., Bouyssie D., Pichereaux C., Purgatto E., Bouzayen M., Latché A., Pech J.C. (2012). Proteomic analysis of chloroplast-to-chromoplast transition in tomato reveals metabolic shifts coupled with disrupted thylakoid biogenesis machinery and elevated energy-production components. Plant Physiol..

[24] Maass D., Arango J., Wüst F., Beyer P., Welsch R. (2009). Carotenoid crystal formation in Arabidopsis and carrot roots caused by increased phytoene synthase protein levels. PLoS ONE.

[25] McGuire R.G. (1992). Reporting of objective color measurements. HortScience.

